# Effective impairment of myeloma cells and their progenitors by blockade of monocarboxylate transportation

**DOI:** 10.18632/oncotarget.5598

**Published:** 2015-09-10

**Authors:** Derek James Hanson, Shingen Nakamura, Ryota Amachi, Masahiro Hiasa, Asuka Oda, Daisuke Tsuji, Kohji Itoh, Takeshi Harada, Kazuki Horikawa, Jumpei Teramachi, Hirokazu Miki, Toshio Matsumoto, Masahiro Abe

**Affiliations:** ^1^ Department of Hematology, Endocrinology and Metabolism, Institute of Biomedical Sciences, Tokushima University Graduate School, Tokushima, Japan; ^2^ Department of Orthodontics and Dentofacial Orthopedics, Institute of Biomedical Sciences, Tokushima University Graduate School, Tokushima, Japan; ^3^ Department of Biomaterials and Bioengineering, Institute of Biomedical Sciences, Tokushima University Graduate School, Tokushima, Japan; ^4^ Division of Bio-imaging, Institute of Biomedical Sciences, Tokushima University Graduate School, Tokushima, Japan; ^5^ Department of Histology and Oral Histology, Institute of Biomedical Sciences, Tokushima University Graduate School, Tokushima, Japan; ^6^ Division of Transfusion Medicine and Cell Therapy, Tokushima University Hospital, Tokushima, Japan

**Keywords:** multiple myeloma, monocarboxylate transporter, lactate, metabolism

## Abstract

Cancer cells robustly expel lactate produced through enhanced glycolysis via monocarboxylate transporters (MCTs) and maintain alkaline intracellular pH. To develop a novel therapeutic strategy against multiple myeloma (MM), which still remains incurable, we explored the impact of perturbing a metabolism via inhibiting MCTs. All MM cells tested constitutively expressed *MCT1* and *MCT4*, and most expressed *MCT2*. Lactate export was substantially suppressed to induce death along with lowering intracellular pH in MM cells by blockade of all three MCT molecules with α-cyano-4-hydroxy cinnamate (CHC) or the MCT1 and MCT2 inhibitor AR-C155858 in combination with *MCT4* knockdown, although only partially by knockdown of each MCT. CHC lowered intracellular pH and severely curtailed lactate secretion even when combined with metformin, which further lowered intracellular pH and enhanced cytotoxicity. Interestingly, an ambient acidic pH markedly enhanced CHC-mediated cytotoxicity, suggesting preferential targeting of MM cells in acidic MM bone lesions. Furthermore, treatment with CHC suppressed hexokinase II expression and ATP production to reduce side populations and colony formation. Finally, CHC caused downregulation of homing receptor CXCR4 and abrogated SDF-1-induced migration. Targeting tumor metabolism by MCT blockade therefore may become an effective therapeutic option for drug-resistant MM cells with elevated glycolysis.

## INTRODUCTION

Multiple myeloma (MM) has a unique propensity to develop and expand almost exclusively in the bone marrow. The bone marrow microenvironment is skewed by MM cells, which underlies the unique pathophysiology of MM and confers aggressiveness and drug resistance [1-7]. The CXCR4/SDF-1 signaling axis has garnered attention for its importance in mediating chemotaxis of malignant cells to these niches [8-11]. Cancer cells expressing CXCR4 metastasize to tissues that highly produce SDF-1 including bone marrow [12]; MM cells express CXCR4 and migrate to the bone marrow niche where interaction with osteoclasts and bone marrow stroma cells enhances MM cell survival and proliferation [4, 9, 10].

Like other cancers, MM cells tend to develop a drug-resistant side population (SP) as defined by breast cancer resistance protein (BCRP)-mediated ability to extrude Hoechst33342 dye [13]. BCRP, an ATP binding cassette (ABC) transporter, expels chemotherapeutic drugs, and its expression is associated with poor prognosis [14]. Thus, the drug-resistant SP persists as an issue to be addressed in the treatment of MM.

Cancer cells exhibit an altered metabotype of reduced oxidative phosphorylation and enhanced glycolysis, even in the presence of adequate oxygen – a phenomenon known as aerobic glycolysis, or the Warburg effect [15-17]. This advantageous phenotype is characterized by upregulation of glycolytic enzymes such as hexokinase II, and can contribute to the enhanced *de novo* synthesis of ATP and anabolic intermediates required for cell growth, while generating important amounts of lactate as a byproduct [18]. Monocarboxylate transporters (MCTs) are passive H^+^-symporters of lactate [19] whose over-expression, with MCT1/4 chaperone CD147, is integral to tumor cells' hyper-glycolytic phenotype [20-23]. Cancer cells are able to maintain alkaline intracellular pH by expelling lactate, contributing to their robust proliferation, while the resulting acidic extracellular microenvironment blunts the anti-tumor effects of local immune cells and chemotherapeutic agents [24-28]. The importance of elevated glycolytic metabolism has been demonstrated in MM cells, highlighting the roles of hexokinase II [29], PDK1 [30, 31] or CD147 [32].

Here, we investigated the impact of MCT blockade on MM cell survival and drug resistance. MCT inhibition decreased lactate export while lowering intracellular pH in MM cells to trigger their death; it also impaired a glycolytic phenotype of MM cells while curtailing ATP production and hexokinase II expression, along with eradicating drug-resistant SP and clonogenic progenitors. MCT inhibition also attenuated CXCR4 expression in MM cells and their chemotaxis towards SDF-1 gradients. These results underscore the value of MCT inhibition for targeting glycolytic drug-resistant MM cells and their progenitors.

## RESULTS

### MCT blockade induces MM cell death

**Figure 1:**
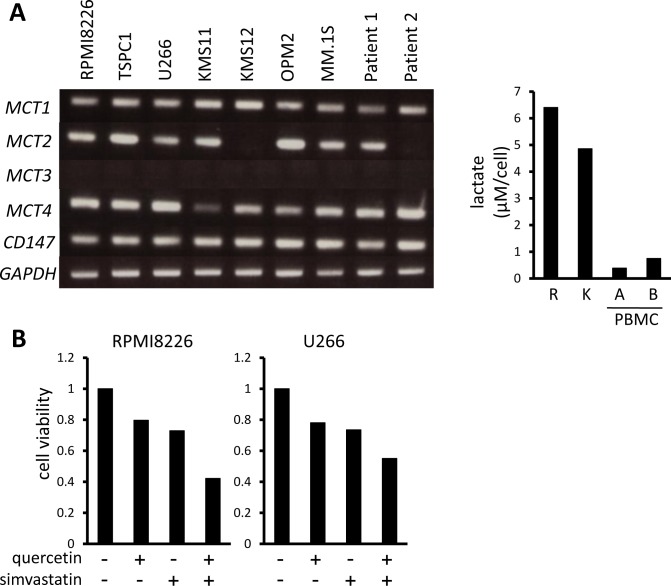

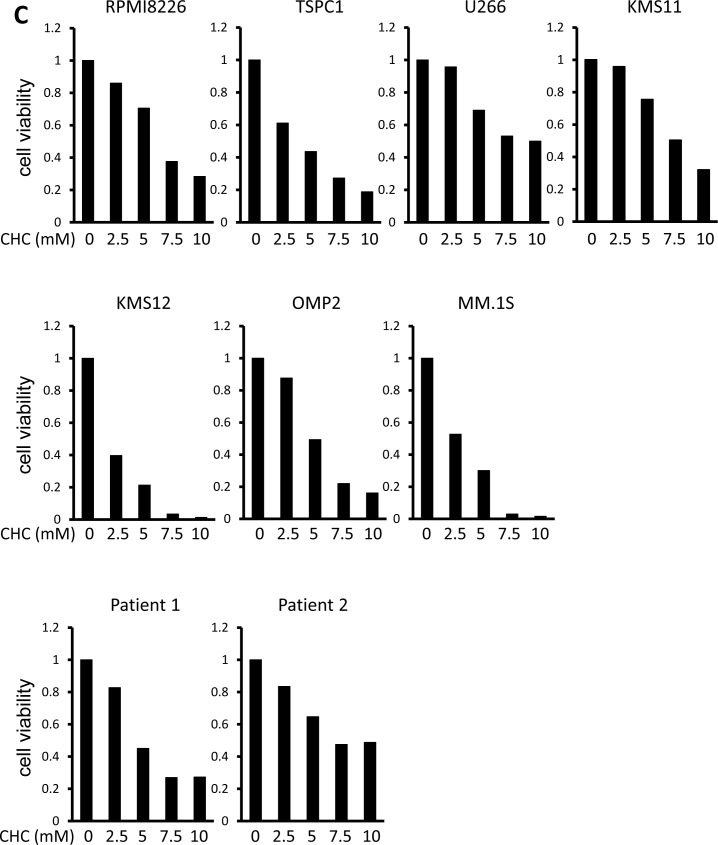
MCT expression and cytotoxic effects of MCT blockade on MM cells **A.** The expression of *MCT1, MCT2, MCT3*, *MCT4* and *CD147* was analyzed by RT-PCR using total RNA isolated from MM cell lines as indicated and primary MM cells from 2 patients with MM (left). *GAPDH* was used as an internal control. RPMI8226 cells (R), KMS11 cells (K), PMBCs from two healthy donors were incubated for three hours, and supernatants were assayed for lactate content (right). Lactate concentrations were divided by cell numbers as counted by trypan blue assay. **B.** MM cell lines were incubated for 24 hours with 50 μM quercetin and/or 10 μM simvastatin and then subjected to a WST8 viability assay. Ratios of viable cells from the baseline were shown. **C.** MM cell lines and primary MM cells were cultured for 24 hours under indicated conditions, then subjected to a WST8 viability assay. Ratios of viable cells from the baseline were shown.

We previously demonstrated that MM cells aberrantly express hexokinase II and have a hyper-glycolytic phenotype to robustly expel lactate [29, 33]. MM cell lines and primary MM cells all constitutively expressed the lactate transporters *MCT1* and *MCT4* as well as their chaperone protein, *CD147*, and most expressed MCT2 (Figure 1A left). Consistent with our previous observation that MM cells are susceptible to inhibition of glycolysis compared to normal cells [29, 33], MM cells produced much more lactate than peripheral blood mononuclear cells from healthy donors (Figure 1A right), supporting MCT molecules as a specific target in MM cells. Because MCT1 and MCT4 are major lactate transporters in cancer cells, we next examined the cytotoxic effects of the MCT1 inhibitor quercetin and MCT4 inhibitor simvastatin on MM cells to determine the role of MCT1 and MCT4 in MM cell survival. Treatment with quercetin or simvastatin alone induced moderate cell death in MM cells, and combined treatment had a greater effect than either agent alone (Figure 1B).

Treatment with α-cyano-4-hydroxy cinnamate (CHC), a known inhibitor of MCT1, MCT2 and MCT4, dose-dependently induced cell death in MM cell lines and primary MM cells (Figure 1C). Thus, monocarboxylate transportation across membranes appears crucial for MM cell survival.

### CHC and metformin cooperatively decrease intracellular pH levels and induce cell death in MM cells

**Figure 2:**
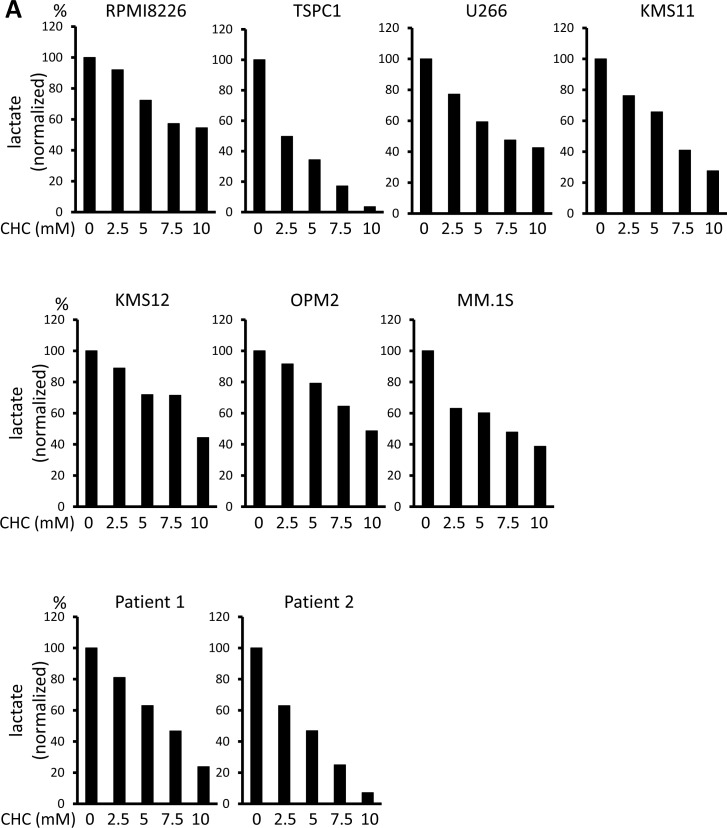

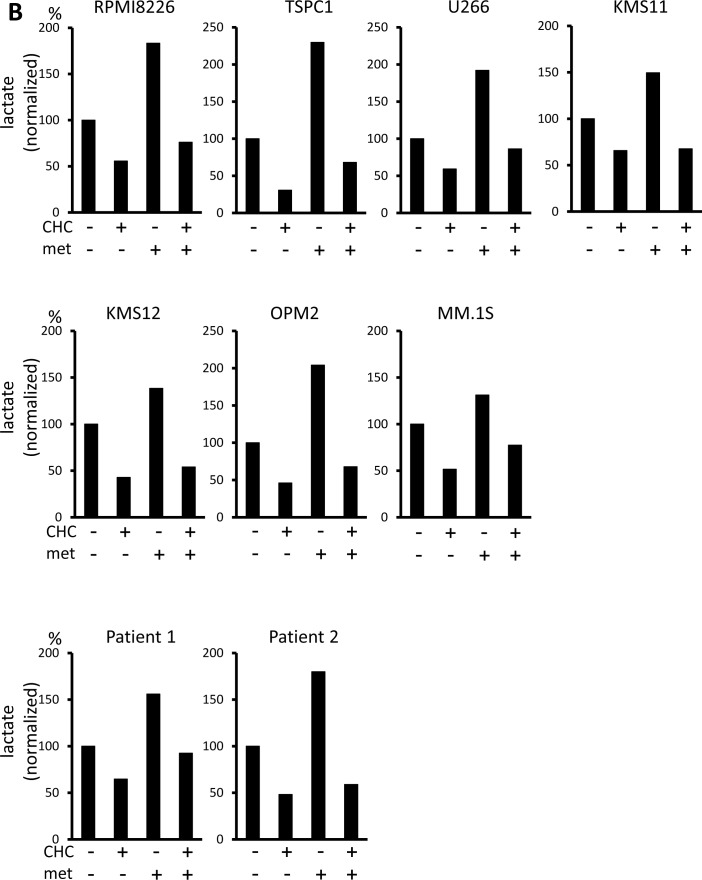

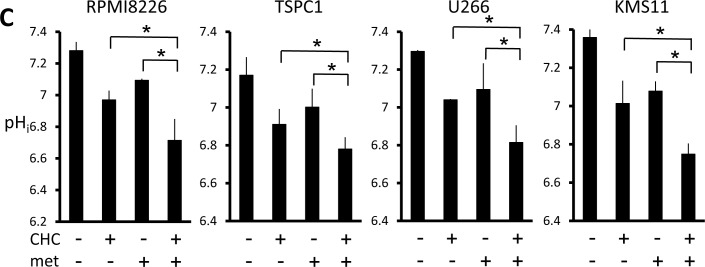

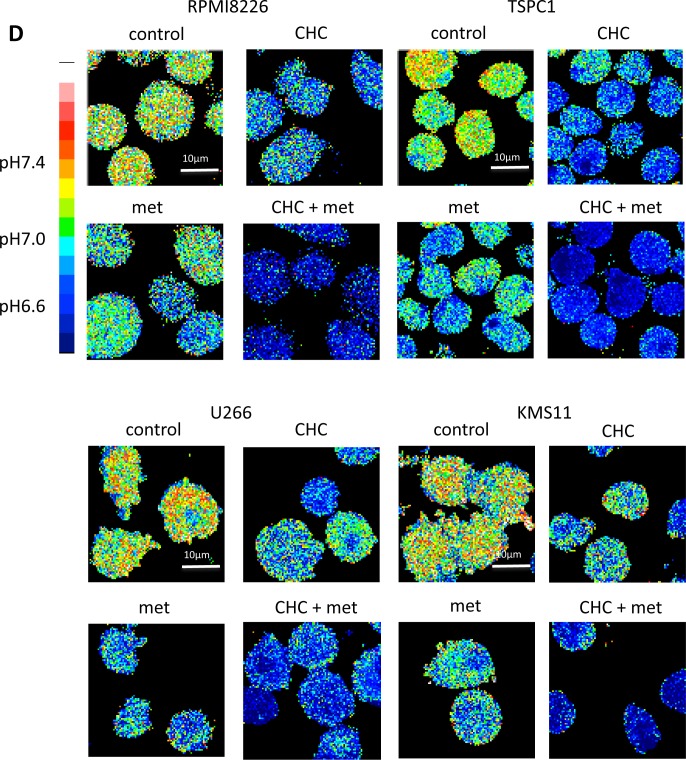

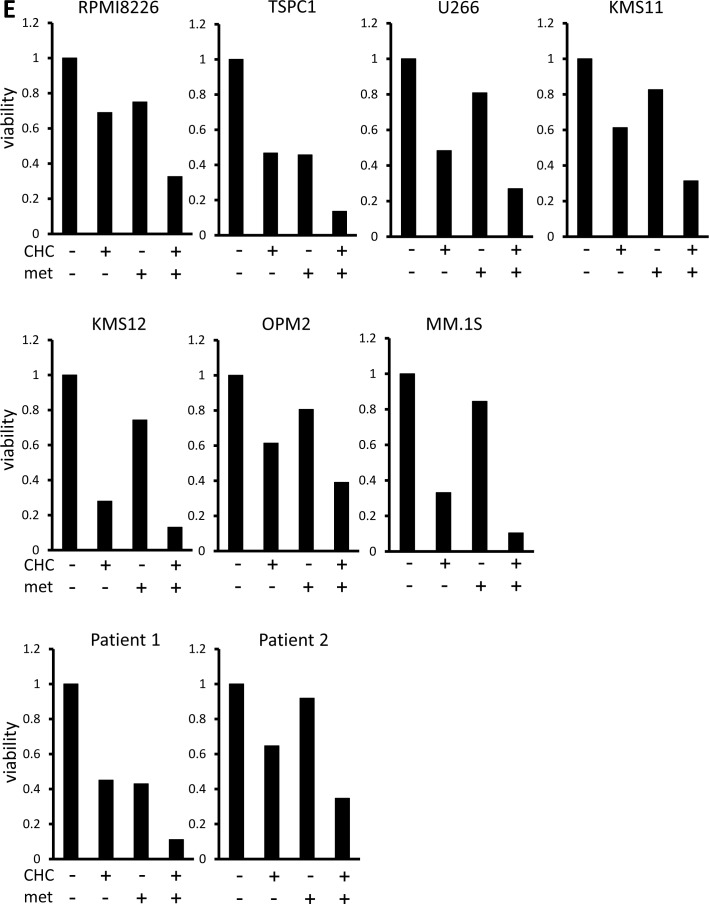

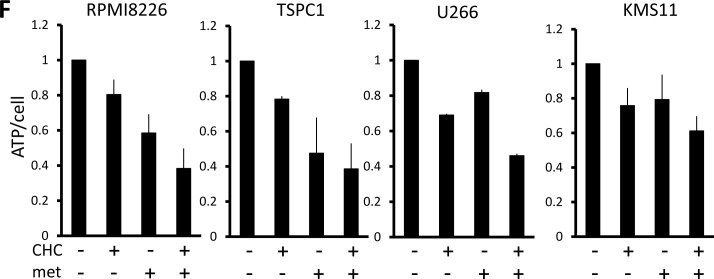
Additive effects of MCT blockade and metformin on intracellular pH and viability in MM cells **A.** MM cell lines and primary MM cells were cultured for three hours under indicated conditions; supernatants were assayed for lactate content. Results were shown as per cent change from baseline. **B.** MM cell lines and primary MM cells were cultured for three hours with 5 mM CHC and/or 10 mM metformin; supernatants were assayed for lactate content. Results were shown as per cent change from baseline. **C.** MM cells were cultured for three hours with 5 mM CHC and/or 10 mM metformin, then stained with BCECF-AM and assessed by spectrophotometer. Ratios of fluorescence intensities were used to calculate intracellular pH (pH_i_) as outlined in Methods. Results from three independent experiments were shown as the mean +/− SD. *p value < 0.05. **D.** Cells were treated the same as in **C.** and then photographed using a fluorescence microscope to produce emission ratio images. **E.** MM cell lines and primary MM cells were cultured for 24 hours with 5 mM CHC and/or 10 mM metformin, then subjected to a WST8 viability assay. Ratios of viable cells from the baseline were shown. **E.** MM cells were cultured for 2 hours with 5 mM CHC and/or 10 mM metformin and assayed for ATP content. Results from three independent experiments were expressed as ratios of change from the baseline with the mean +/− SD.

Lactate is an MCT substrate that is pivotal to energy and biomass metabolism as well as pH homeostasis of cancer cells. We next explored the effect of CHC treatment on pH levels in MM cells. CHC dose-dependently reduced lactate concentrations in medium supernatants of MM cell cultures, indicating curtailed lactate export (Figure 2A). Metformin, a stimulator of glycolysis and lactate production, drastically increased extracellular lactate concentration above control levels, but this was reversed by combination with CHC (Figure 2B), showing effective blockage of lactate export even in MM cells with increased lactate production. To check for intracellular acidification, spectrophotometer measurements were performed using the pH indicator dye BCECF-AM, which permeates into cells where cellular esterases cleave the acetoxymethyl groups, thereby enabling pH-dependent fluorescence in the cytoplasm. Consistent with the lactate transport blockade and concomitant intracellular lactate buildup, or CHC treatment depressed intracellular pH below control levels; combination with metformin enhanced this effect (Figure 2C). These results were further confirmed by photographing individual cells under a fluorescence microscope under the same treatment conditions with BCECF-AM as used in spectrophotometer experiments (Figure 2D). These data suggested that CHC treatment depressed intracellular pH by lactate sequestration in MM cells and that combined treatment with metformin exacerbated intracellular acidification. At concentrations that were moderately cytotoxic in single treatment, CHC and metformin induced additive cell death in MM cells (Figure 2E), correlating with intracellular acidification responses [34].

Cancer cells produce ATP for their growth and survival largely through enhanced glycolysis. In consideration that CHC and metformin block different outlets of the glycolytic pathway (MCT inhibition and oxidative phosphorylation inhibition, respectively), their individual and combined effects on MM cell energetics were assessed. Single treatment with CHC or metformin lowered ATP levels in all cell lines within two hours, and the two cooperatively lowered ATP levels (Figure 2F). The combinatory treatment was able to perturb metabolisms in MM cells along with intracellular acidification, which effectively induced energy crisis in MM cells.

### Simultaneous inhibition of MCT1, MCT2, and MCT4 curtails lactate transport and viability in MM cells

**Figure 3:**
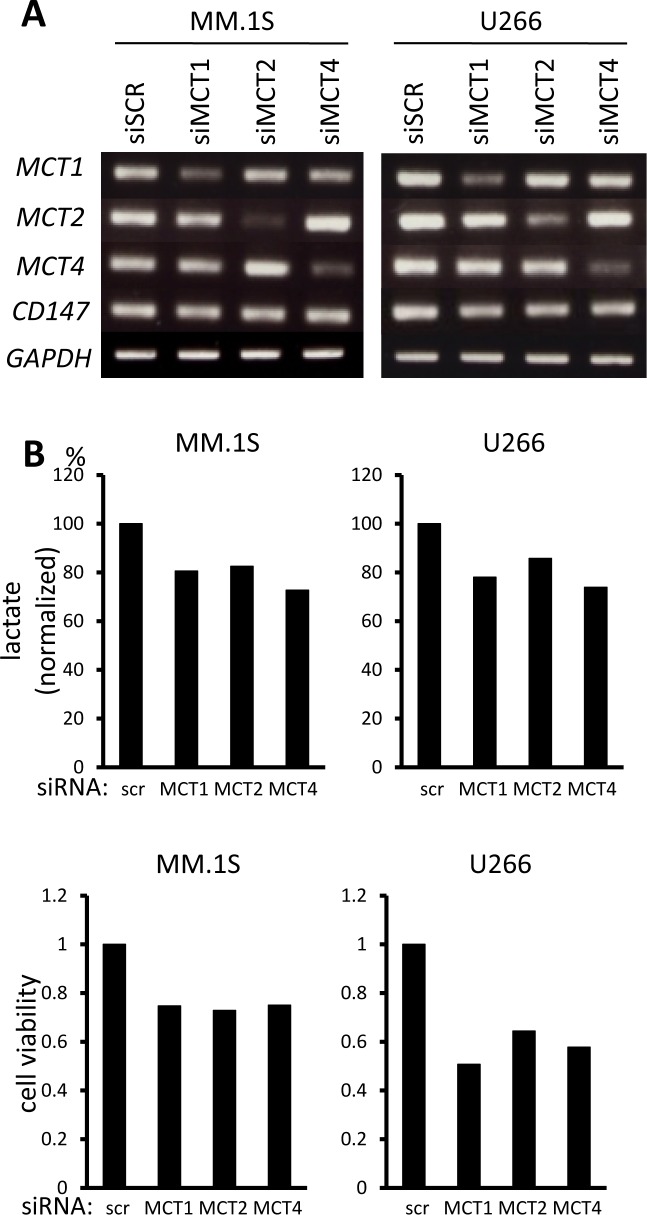

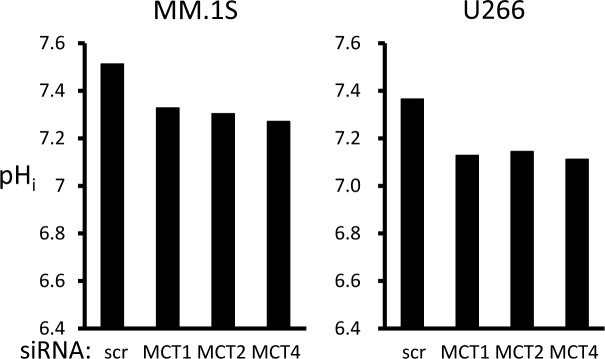

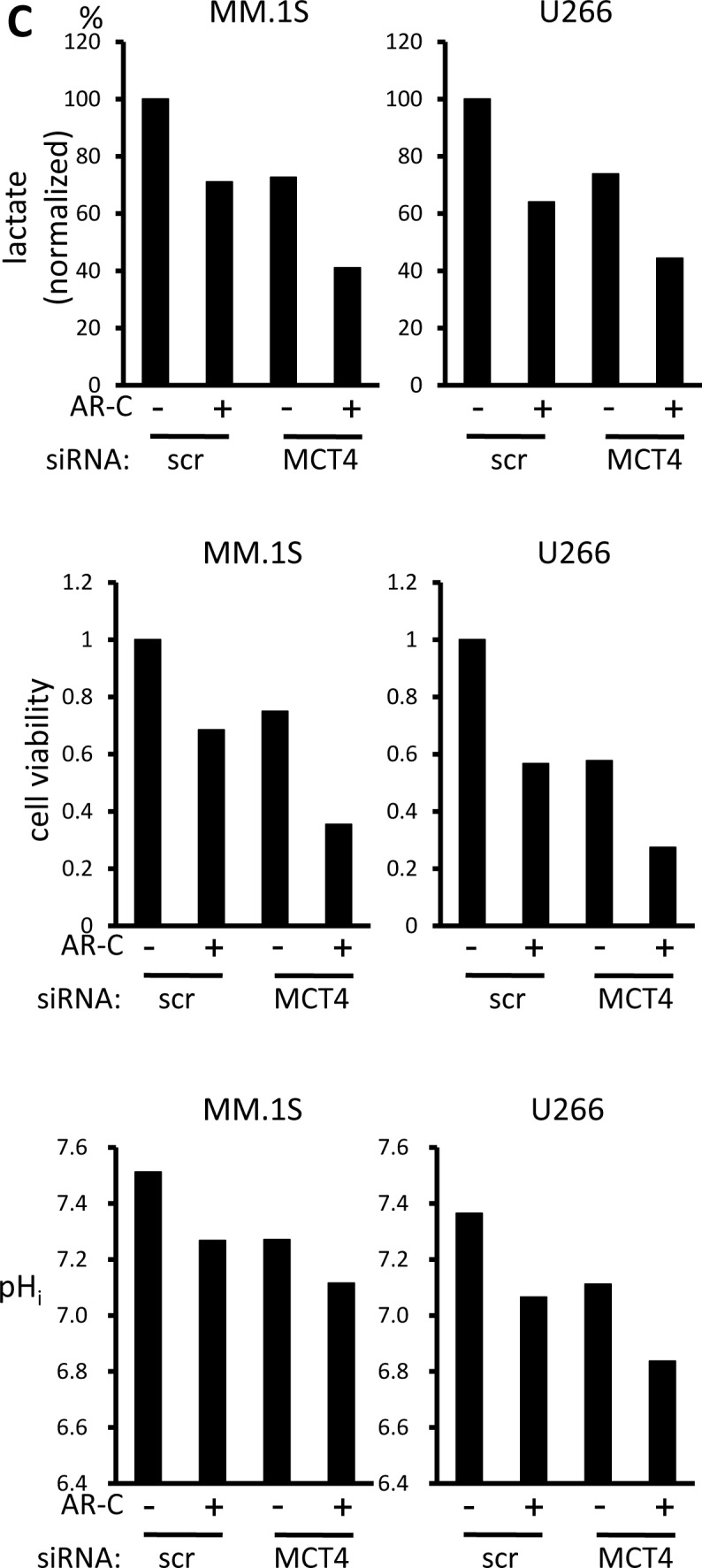
Contribution of individual MCT molecules to lactate export and survival in MM cells **A.** The expression of *MCT1, MCT2, MCT4* and *CD147* was analyzed by RT-PCR results using total RNA isolated from MM.1S and U266 cells that were transiently transfected with scramble siRNA (siSCR) or the indicated siRNA for 14 hours. **B.** The aliquots of MM cells transfected with siRNA for 14 hours in (A) were washed in PBS and cultured for three hours; supernatants were assayed for lactate content (upper). Results were shown as per cent change from baseline. The aliquots of MM cells in (A) were cultured for an additional 24 hours, then subjected to a WST8 viability assay (middle). Ratios of viable cells from the baseline are shown. The aliquots were also cultured for three hours, then intracellular pH (pH_i_) was assessed by spectrophotometer after staining with BCECF-AM (lower). Ratios of fluorescence intensities were used to calculate pH_i_. **C.** Aliquots of MM.1S and U266 cells transfected with scramble siRNA (siSCR) or *MCT4* siRNA for 14 hours were cultured in the presence or absence of 300 nM AR-C155858. After culturing for three hours, supernatants were assayed for lactate content (upper). Results are shown as per cent change from the baseline. After culturing for 24 hours, the cells were subjected to a WST8 viability assay (middle). Ratios of viable cells from the baseline are shown. After culturing for three hours, intracellular pH (pH_i_) was assessed by spectrophotometer after staining with BCECF-AM (lower). Ratios of fluorescence intensities were used to calculate pH_i_.

In order to clarify the role of individual MCT molecules on lactate export in MM cells, we looked at the effects of siRNA knockdown of individual MCT molecules. siRNA was successfully transfected to knockdown *MCT1*, *MCT2*, or *MCT4* in MM.1S and U266 cells (Figure 3A). Single knockdown of *MCT1*, *MCT2*, or *MCT4* partially decreased lactate export, viable cell counts and intracellular pH, suggesting that each of these molecules makes a contribution to MM cell metabolism and homeostasis (Figure 3B). To further confirm the effects of simultaneously inhibiting MCT1, MCT2 and MCT4, we examined using AR-C155858, an MCT1 and MCT2 inhibitor, and siRNA knockdown of *MCT4*, because CHC inhibits a mitochondrial pyruvate transporter as well [19]. Treatment with AR-C155858 partially suppressed lactate export and induced death along with lowing intracellular pH in MM.1S and U266 cells; knockdown of *MCT4* by siRNA substantially enhanced these effects mediated by AR-C155858 (Figure 3C). Taken together, these results demonstrated the importance of inhibiting all three MCT molecules when targeting MM cells.

### Induction of MM cell death is potentiated in acidic conditions

**Figure 4:**
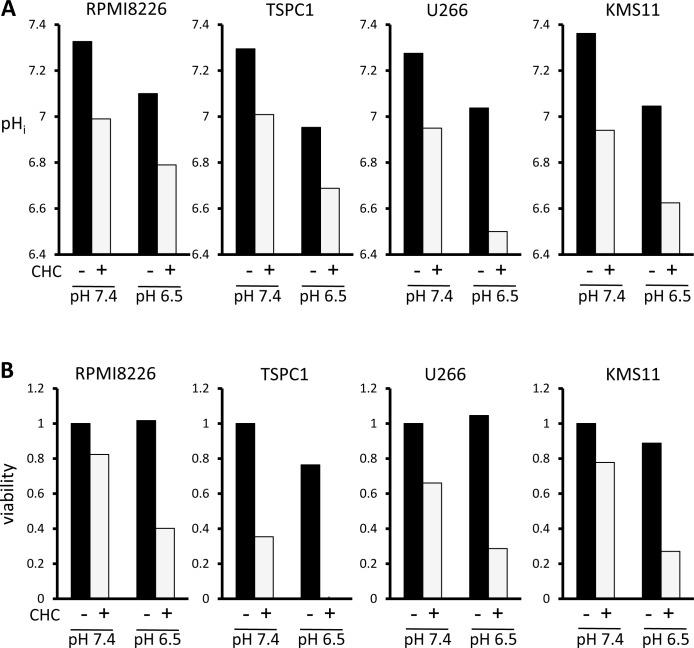
Influence of extracellular pH on intracellular acidification and cytotoxic effects of MCT blockade on MM cells **A.** MM cells were cultured for three hours at pH 7.4 or pH 6.5 with 5 mM CHC, then stained with BCECF-AM and assessed by spectrophotometer. Ratios of fluorescence intensities were used to calculate intracellular pH (pH_i_) as outlined in Methods. **B.** MM cells were cultured for 24 hours with or without 5 mM CHC at pH 7.4 or pH 6.5 and then subjected to a WST8 viability assay. Ratios of viable cells from the baseline were shown.

Compared to normal tissue, tumors tend to develop abnormally acidic microenvironments, in the pH 6.5 range, which confer immune evasion capability and enhanced metastatic aggressiveness [24-28]. The ambient acidified pH of tumor microenvironments in acidic osteolytic lesions in MM might present an opportunity to achieve favorable therapeutic window; therefore, the cytotoxic effect of MCT blockade on MM cells was assessed at tumor-like pH vis-à-vis physiological pH. When MM cells were cultured at pH 6.5, intracellular pH levels were lowered (Figure 4A), but viability was only marginally affected in RPMI8226, U266 and KMS11 cells (Figure 4B). However, treatment with CHC further depressed intracellular pH in MM cells cultured at pH 6.5 to markedly enhance MM cell death compared to culturing at pH 7.4. Thus, while tumor-like extracellular pH 6.5 alone did not induce considerable cell death, it appreciably lowered intracellular pH and markedly enhanced CHC-mediated cytotoxicity in MM cells. These results suggest that extracellular as well as intracellular pH plays a role in the cytotoxic effect of CHC.

### CHC treatment reduces SP as well as colony-forming populations in MM cells

**Figure 5:**
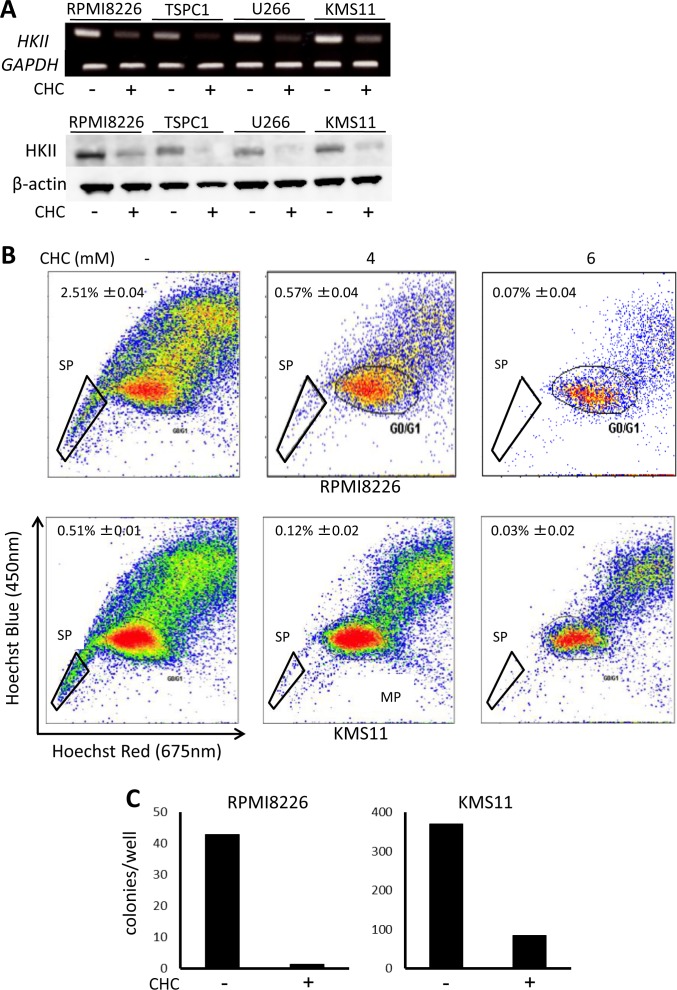
Effects of MCT blockade on hexokinase II expression and MM progenitors **A.** MM cells were cultured for 16 hours with or without 5 mM CHC and then subjected to RT-PCR or Western blot analysis. *GAPDH* or β-actin was used as an internal control. **B.** RPMI8226 or KMS11 cells were cultured for 24 hours with or without CHC as indicated, stained with Hoechst33342, and then analyzed by flow cytometry. **C.** RPMI8226 or KMS11 cells were cultured for 24 hours with or without 6 mM CHC and plated in nitrocellulose media for 14 days. Colony numbers were counted.

A persistent issue in MM is effective targeting of drug-resistant MM cells or MM progenitors, which is associated with patient relapse and poor prognosis; new avenues to address this would be beneficial. We previously demonstrated that SP is a highly glycolytic fraction in MM cells, and that inhibition of glycolysis preferentially targets and reduces a SP fraction of MM cells and clonogenic MM cells with colony formation [29]. Hexokinase II is aberrantly overexpressed in cancer cells and mediates the irreversible first step of glycolysis; it contributes to the Warburg effect and is thought to prevent pro-apoptotic proteins Bax and Bad from provoking cytochrome *c* release in cancer cells, making it a metabolic target of interest [35]. CHC treatment markedly reduced hexokinase II expression in MM cells at mRNA and protein levels (Figure 5A). Thus, treatment with CHC substantially reduced the crucial glycolytic machinery hexokinase II in MM cells. Together with ATP reduction by treatment with CHC, CHC appears to target glycolysis enhanced in MM cells. Importantly, CHC treatment nearly eradicated the SP fractions of both RPMI8226 and KMS11 cells (Figure 5B), and also drastically reduced their colony formation (Figure 5C), indicating effectiveness against clonogenic MM cell populations implicated in patient relapse.

### CHC treatment attenuates CXCR4 expression and chemotactic capabilities of MM cells

**Figure 6:**
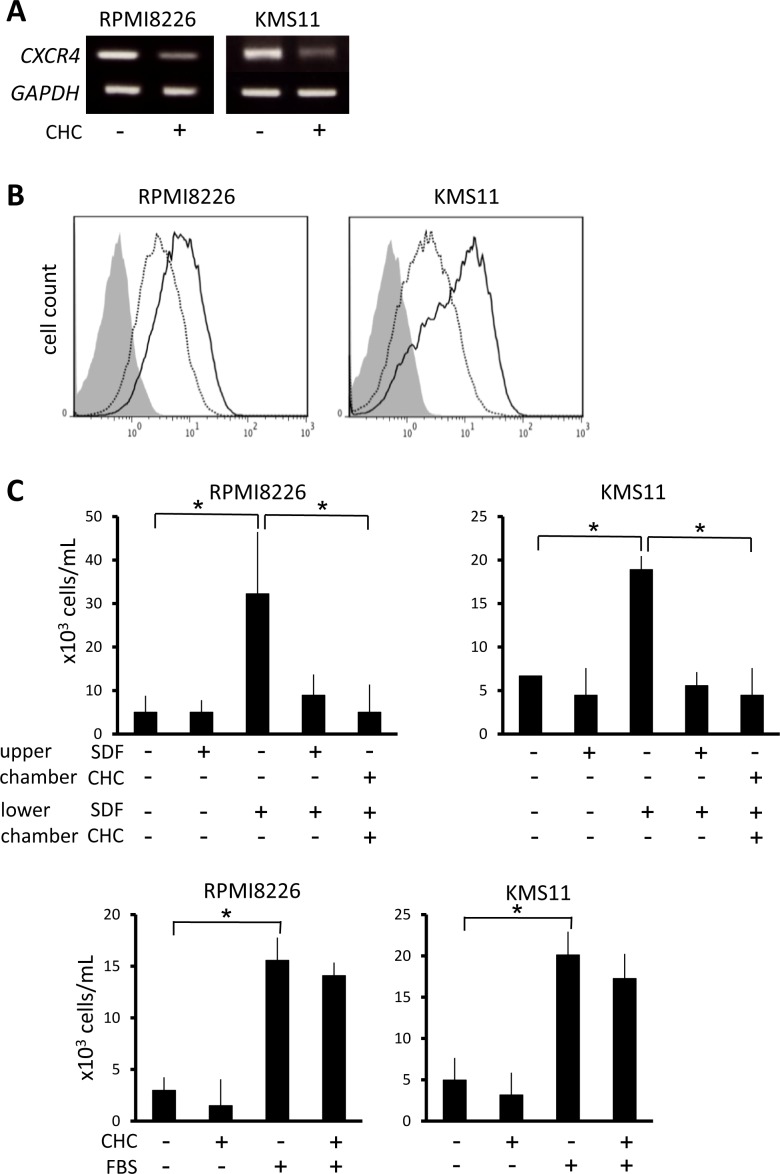
Suppression of CXCR4 expression and migration of MM cells by MCT blockade **A.** MM cells were incubated for 16 hours with or without 5 mM CHC and then subjected to RT-PCR. **B.** MM cells were cultured in the presence (dotted line) or absence (solid line) of 4 mM CHC for 24 hours, stained with phycoerythrin-labeled anti-CXCR4 antibody, and then subjected to flow cytometry. Background staining with control IgG was shown in gray. **C.** MM cells were plated in upper chambers with 8-μm pore filters; rhSDF-1α (final concentration 30 nM), CHC (final concentration 5 mM) or FBS (final concentration 10%) was added as indicated. After 24 hours, cells that had migrated to the lower chambers were counted. Results from three independent experiments were shown as the mean +/− SD. *p value < 0.05.

CXCR4 signaling potentiates homing of MM cells up SDF-1 gradients to bone marrow microenvironments that favor their survival and proliferation, and this has attracted attention as a possible therapeutic target in MM treatment [36]. In RPMI8226 and KMS11 cells, CHC treatment attenuated CXCR4 at mRNA and surface protein levels (Figure 6A and 6B). Whereas rhSDF-1α induced substantial migration of RPMI8226 and KMS11 cells, the presence of CHC along with rhSDF-1α resulted in migration activity similar to controls that lacked rhSDF-1α (Figure 6C). However, CHC only marginally affected the migration of MM cells towards FBS (Figure 6C), consistent with the hypothesis that the effect on migration is mediated through disruption of the rhSDF-1α pathway, per se.

## DISCUSSION

It has been well established that aberrant metabolism is one important phenotypic trait of cancer cells distinguishing them from normal tissues and therefore represents an attractive potential target with favorable therapeutic index. The Warburg effect crucially contributes to tumor biomass production and is such a reliable hallmark of malignancy that it is the basis of the diagnostic fluorodeoxyglucose positron emission tomography (FDG-PET) scan. Accordingly, MCT expression is known to be upregulated in cancer cells to export lactate, a byproduct of the Warburg effect, and lactate accumulation in tumor milieus is associated with poor clinical outcomes [18, 27, 37]. Secretion of excess lactate by cancer cells contributes to acidosis of the tumor microenvironment, which blunts anti-tumor immune responses [24-26]. MM cells constitutively expressed *MCT1* and *MCT4*, and most expressed *MCT2* (Figure 1A). Single knockdown of *MCT1*, *MCT2*, or *MCT4* by siRNA only slightly impaired MM cell survival and function; however, blockade of all three of these molecules is most effective when targeting MM cells (Figures 2 and 3).

Induction of intracellular acidosis correlated with induction of cell death under treatment with CHC and/or metformin (Figure 2), in agreement with previous reports showing lowered intracellular pH to be conducive to apoptosis triggering [38-42]. Furtheremore, tumor-like extracellular acidic pH markedly enhanced CHC-mediated cytotoxicity in MM cells. Therefore, MCT blockade is suggested to be able to preferentially target tumor cells endowed with drug resistance in lactate-abundant acidic milieu or acidic bone-resorbing lesions in MM.

CHC-mediated attenuation of hexokinase II protein levels and ATP levels suggests impairment of glycolytic flux, an important source of energy and biomass in cancer cells. Since intracellular acidification occurred acutely, it is possible that CHC-induced lactate buildup plateaus at or above a threshold where it may cause feedback inhibition of glygolitic machinery [43]. Furthermore, hexokinase II supports cell survival by inhibiting formation of the permiability transition pore in the outer mitochondrial membrane and preventing release of apoptosis-inducing cytochrome *c* [35, 44]. Thus, various factors including intracellular acidification, impaired energy production, and loss of anti-apoptotic hexokinase II may contribute to the mechanism of how CHC caused cell death in MM cells and their progenitors.

The upregulation of BCRP expression and function is a mechanism responsible for formation of the clonogenic SP and development of drug resistance. Considering the aforementioned effect of CHC on MM cell energy levels and hexokinase II expression, it is plausible that CHC-induced ATP paucity contributes to reduced (ATP-dependent) BCRP function under these treatments, as suggested by our previous work showing that inhibition of glycolysis by 3-bromo-pyruvate suppresses ATP production and restores drug sensitivity in BCRP-expressing RPMI8226 cells as well as p-glycoprotein-expressing KG1 leukemia and HepG2 hepatoma cells [29].

Inhibition of MCTs in MM cells attenuated CXCR4 expression and potently suppressed rhSDF-1α-induced migration in MM cells (Figure 6), implying that intracellular acidification impairs the CXCR4/SDF-1 signaling axis. If such an approach could disrupt MM cell homing to the bone marrow niche *in vivo*, it may deprive them of growth and survival signals provided by osteoclasts and bone marrow stromal cells and enhance their vulnerability to chemotherapeutic agents.

Finally, alternative approaches to disrupting lactate transport in MM cells are also possible. For example, monoclonal antibody directed against CD147 can inhibit its association with MCT1 to curtail lactate transport and reduce intracellular pH, ATP levels, and viability in cancer cells [22]. The important contribution of CD147 to lactate export [32] and cell proliferation [45] was also recently shown in MM cells, suggesting the possibility of such an approach for metabolic disruption in MM cells as well.

The present work has shown that targeting the glycolytic phenotype of MM cells by blockade of MCTs potently effects cytotoxicity against drug-resistant, lactate-expelling MM cells and their progenitors. This approach also decreased CXCR4 expression and migration in MM cells. These results are consistent with recent investigations aimed at cancer metabolism and point to the promise of such approaches for novel treatment modalities. Further translational research will elucidate the dividends these may yield in improved clinical outcomes.

## MATERIALS AND METHODS

### Reagents

Reagents were purchased as follows: CHC, metformin, simvastatin, quercetin and mouse anti-β-actin antibody from Sigma-Aldrich (St. Louis, MO); AR-C155858 from Tocris Bioscience (Bristol, UK); BCECF-AM from Dojindo (Kumamoto, Japan); nigericin from Cayman Chemical (Ann Arbor, MI); horseradish-peroxidase-conjugated donkey anti-goat IgG and horse anti-mouse IgG from Cell Signaling Technology (Beverly, MA); goat anti-human hexokinase II polyclonal antibody from Santa Cruz Biotechnology (Dallas, TX); phycoerythrin-conjugate of mouse IgG antibody and phycoerythrin-conjugate of mouse anti-human CXCR4 monoclonal antibody from BioLegend (San Diego, CA); rhSDF-1α from R&D systems (Minneapolis, MN).

### Cells and culture

The human MM cell lines RPMI8226, U266 and KMS11 were obtained from the American Type Culture Collection (ATCC, Rockville, MD); OPM2 from the German Collection of Microorganisms and Cell Cultures (Braunschweig, Germany); and KMS12-BM from the Health Science Resources Bank. MM.1S was kindly provided by Dr. Steven Rosen (Northwestern University, Chicago, IL, USA). TSPC-1 MM cell line was established in our laboratory [5]. MM cells were purified from bone marrow mononuclear cells from patients with MM by positive selection using CD138 microbeads and Miltenyi magnetic cell sorting system (Miltenyi Biotec, Auburn, CA) according to the manufacture's instruction. Peripheral blood mononuclear cells were isolated as previously reported [6]. Cells were cultured in RPMI1640 medium with 10% FBS, penicillin G (50 μg/ml) and streptomycin (50 μg/ml). In experiments using CHC, medium pH was adjusted to pH 7.4 unless otherwise indicated. All procedures involving human specimens were performed with written informed consent according to the Declaration of Helsinki and using a protocol approved by the Institutional Review Board for human protection.

### Evaluation of cell viability

Cell viability was determined by Cell Counting Kit-8 assay (Dojindo, Kumamoto, Japan) according to the manufacture's instructions. Briefly, cells were plated in a 96-well plate and incubated with 2-(2-methoxy-4-nitrophenyl)-3-(4-nitrophenyl)-5-(2,4-disulphophenyl)-2H-tetrazolium monosodium salt (WST-8) for 1–4 hours. The absorbance of each well was measured at 450 nm with iMark™ microplate reader (Bio-Rad Laboratories, Hercules, CA). To confirm results, viable cell numbers were also counted by a trypan blue dye exclusion assay as we previously described [6].

### RT-PCR

Total RNA was extracted using Tri Reagent according the manufacturer's instructions and then converted to cDNA libraries using SuperScript II Reverse Transcriptase (Invitrogen, Carlsbad, CA) with random primers (Promega, Madison, WI). Sequences of specific primers (Invitrogen, Carlsbad, CA) for PCR reactions were as follows: *MCT1* sense, 5′-CCATTGTGGAATGCTGTCCT-3′; *MCT1* antisense, 5′-CCACATGCCCAGTATGTGTA-3′; *MCT2* sense, 5′-GGCCCTCCTCTTGCAGGTAA-3′; *MCT2* antisense, 5′-CAATGAGCAGCCACACGCTT-3′; *MCT3* sense, 5′-GAAGGAGACTTGGGAGGCAGC-3′; *MCT3* antisense, 5′-AGAAGACGCTCACGGCTTTG-3′; *MCT4* sense, 5′-ATCCTGGGCTTCATTGACAT-3′; *MCT4* antisense, 5′-ATGGAGAAGCTGAAGAGGTA-3′; *CD147* sense, 5′-TCCTGGATGATGACGACGCC-3′; *CD147* antisense, 5′-AAGAGTTCCTCTGGCGGACG-3′; *CXCR4* sense, 5′-TTTCTTCGCCTGTTGGCTGC-3′; *CXCR4* antisense, 5′-AAGCTAGGGCCTCGGTGATG-3′; *HKII* sense, 5′-TGGAGGGACCAACTTCCGTGTGCT-3′; *HKII* antisense, 5′-TCAAACAGCTGGGTGCCACTGC-3′; *GAPDH* sense, 5′-TGTCTTCACCACCATGGAGAAGG-3′; *GAPDH* antisense, 5′-GTGGATGCAGGGATGATGTTCTG-3′. PCR reactions were carried out using EX Taq hot start polymerase (TaKaRa, Otsu, Japan) with 30 cycles of 30 seconds at 95°C for denaturing, 30 seconds at 58°C for annealing, and 10 seconds at 72°C for elongation; 23 cycles of the same were used for housekeeping gene *GAPDH*. PCR products were then subjected to electrophoresis on ethidium bromide-stained 1% agarose gels and visualized in a Printgraph UV transluminator (ATTO, Tokyo, Japan).

### Lactate measurement

Cells were washed twice with PBS and seeded in a 24-well plate with 1 mL of FBS-free RPMI1640 medium and 4 × 10^5^ cells per well. After three hours cell numbers were counted with trypan blue stain and then centrifuged at 4,000 rpm for five minutes. Supernatants were assayed for lactate concentration using Lactate Assay Kit (BioVision Incorporated, Milpatas, CA) with iMark™ microplate reader (Bio-Rad, Hercules, CA).

### ATP assay

Cells were cultured for two hours in 100-μL aliquots at 4 × 10^5^ cells/mL. After one thorough washing with PBS, the ATP level of each aliquot was measured with a CellTiter Glo ATP assay kit (Promega, Madison, WI) according to manufacturer's instructions; luminescence levels were measured by a microplate reader (Therm Fisher Varioskan Flash; Waltham, MA).

### Intracellular pH measurement

Cells (2 × 10^7^ cells/mL) were cultured without CO_2_ injection at 37°C in HEPES buffer for three hours, followed by staining with BCECF-AM (final concentration 1 μM) for 10 minutes. Cells were washed and resuspended in HEPES buffer, and then fluorescence intensities at wavelength 525 nm were measured by a spectrophotometer (Hitachi, Tokyo, Japan) with excitation wavelength either 488 nm or 439 nm (isosbestic point). The ratio of the two fluorescence intensities was calculated and compared to a calibration curve to calculate absolute pH value. A calibration curve was constructed using ratios of fluorescence intensity values obtained from cells immediately after 10-minute incubation in pH-adjusted high-potassium Na-MOPS calibration buffer (AppliChem, Darmstadt, Germany) plus nigericin (final concentration 10 μg/mL).

### Intracellular pH imaging

Cells (1 × 10^6^ cells/mL) were cultured for three hours, followed by staining with BCECF-AM (final concentration 1 μM). Cells were washed, resuspended in HEPES buffer, and photographed using a fluorescence microscope (Nikon, Tokyo, Japan) with emission wavelength 525/50 nm (range of 500 nm to 550 nm) and excitation wavelength either 488 nm or 405 nm. Color-coded ratio images were produced from this data to show pH change.

### Colony formation assay

RPMI8226 or KMS11 cells were cultured in the presence or absence of CHC for 24 hours. The cells were plated out into a H4034 methylcellulose medium (Stem Cell Technologies, Vancouver, Canada) in triplicate for 14 days. The number of colonies was counted.

### Migration assay

Membrane filters with8-μm pores (Chemotaxicell, Tokyo, Japan) were placedonto24-well culture plates. MM cells were plated outat3 × 10^6^ cells/mL suspended in FBS-free mediumontoupper chambers; the lower chambers were filled with FBS-free medium containing no cells. In positive controls, FBS was added at 10%in lower chambers. After 24 hours, the cell number in the lower chambers was counted.

### Flow cytometry

Cell preparation and staining for flow cytometry were performed as described previously [6]. Approximately 1 × 10^6^ cells were incubated in 100 μL PBS with 2% human γ-globulin with a phycoerythrin-conjugated monoclonal antibody on ice for 40 minutes and then washed. Samples were analyzed by flow cytometry using EPICS-Profile (Coulter Electronics, Hialeah, FL).

### SP analysis

A SP analysis was performed as previously described [46]. Briefly, cells were incubated with 5 μg/mL Hoechst33342 (Sigma-Aldrich, St. Louis, MO) for 90 minutes at 37°C in PBS containing 3% FBS in the presence or absence of 100 μM verapamil (Sigma-Aldrich, St. Louis, MO). Then, the cells were washed and incubated with propidium iodide (1 μg/mL) to discriminate dead cells. SP fractions were analyzed by flow cytometer (Beckman Coulter, Tokyo, Japan).

### Western blotting

Cells were collected and lysed in lysis buffer (Cell Signaling, Beverly, MA) supplemented with 1 mM phenylmethylsulfonyl fluoride and protease inhibitor cocktail solution (Sigma-Aldrich, St. Louis, MO). Cell lysates were electrophoresed in 10% SDS-PAGE gel and blotted onto polyvinylidene difluoride membranes (Millipore, Bedford, MA). After blocking with 5% non-fat dry milk, the membranes were incubated with primary antibodies overnight at 4°C, followed by washing and addition of a horseradish-peroxidase-conjugated secondary antibody for one hour. The protein bands were visualized with an Enhanced Chemiluminescence Plus Western Blotting Detection System (Amersham Biosciences, Piscataway, NJ).

### Small interfering RNA (siRNA) transfection

Small Interfering RNAs (siRNAs) were purchased from Invitrogen (Stealth Select RNAi). The sequences of oligonucleotides synthesized for templates are as follow: MCT1 siRNA, 5′-CAGCAGUAUCCUGGUGAAUAAAUAU-3′ and 5′-AUAUUUAUUCACCAGGAUACUGCUG-3′; MCT2 siRNA, 5′-GAAAUGUCAUUAUGUUCCUAGGUUU-3′ and 5′-AAACCUAGGACCAUAAUGACAUUUC-3′; MCT4 siRNA, 5′-CGGGCCCUACUCCGUCUACCUCUUCA-3′ and 5′-UGAAGAGGUAGACGGAGUAGGGCCG-3′. A concentration of 200 nM of each siRNA was transfected into cells (2×10^6^ cells) by electroporation using a Human Nucleofector Kit (Lonza, Basel, Switzerland) according to the manufacturer's protocol.

### Statistical analysis

Significance was determined by ANOVA with p value of 0.05 considered the threshold for significance.
